# Effects of the homopolymer molecular weight on a diblock copolymer in a 3D spherical confinement

**DOI:** 10.1186/s13065-019-0541-7

**Published:** 2019-02-14

**Authors:** Dung Q. Ly, Charalampos Makatsoris

**Affiliations:** 1 School of Physical Sciences and Computing, University of Central Lancashire, Preston, UK; 2 School of Aerospace, Transportation and Manufacturing, Cranfield University, Cranfield, UK

## Abstract

The morphologies of a diblock copolymer spherically confined within a homopolymer were investigated by using the static self-consistent field theory method. A homogeneous *A*-*B* diblock copolymer sphere was surrounded by a homopolymer *C*. Upon changing the diblock volume fraction, homopolymer molecular weight and the interaction between the copolymer and its surrounding environment, different morphologies of the sphere were observed. Our calculations confirmed that when the homopolymer molecular weight was high a complete macrophase separation between the copolymer and the homopolymer was obtained. However, when the homopolymer molecular weight was low the homopolymer penetrated into the copolymer microdomains, diluting the diblock copolymer and reduced the interaction between the diblock copolymer segments and hence preventing them from segregating.

## Introduction

The quest of creating new functional materials in nanometer scales with targeted properties has attracted much attention from the scientific community in the last two decades [1, 2]. Block copolymers of soft materials have been identified as excellent candidates to fabricate advanced functional materials such as nanoparticles, nanocontainers/nanocapsules, nanowires and nanopores. Recently many efforts have been made to use block copolymers as nanocontainers which can be used as drug delivery vehicles and nanoreactors [3, 4], or as scaffords to position nanoparticles into arrays for applications such as photovoltaic, fuel cells and high-density magnetic storage media [5, 6]. One of the most interesting properties of block copolymers, which are composed of chemically different homopolymers covalently connect at one end, is their ability to self assemble into ordered microdomain structures. The self assembly process is driven by an unfavourable mixing enthalpy coupled with a small mixing entropy, with the covalent bond connecting the blocks preventing macroscopic phase separation. Understanding the behaviour of morphologies of block copolymers under different conditions such as polymeric chain architectures, composition, concentration of solvents, external fields, etc, has attracted a considerable attention. In bulk, depending on the volume fraction of individual blocks, *f*, and a combination of $\chi N$, where $\chi$ is the Flory–Huggins segmental interaction parameter that is inversely proportional to the temperature, and *N* is the degree of polymerization, different structures were observed such as lamellar, cylinder, gyroid and sphere [7, 8]. In confinements, more morphologies which are not formed in the bulk have been obtained when block copolymers are confined between walls in 1D, grafted to surfaces or in a cylindrical pore in 2D [9–12]. In our previous work, by changing the film thickness and surface fields, different phases from wetting layer to parallel cylinder, perpendicular cylinder, perforated lamellar, lamellar, coexisting sphere and lamellar, and coexisting cylinder and lamellar of a triblock copolymer confined between two hard walls were obtained [9]. The effect of a spherical surface on a diblock copolymer which has one end of a polymer chain fixed at the spherical surface was also investigated computationally by Vorselaars et al. [11]. By increasing the volume fraction between the two blocks they observed a sequence of morphologies from dots to stripes then to a layer with holes and finally to a uniform shell [11]. In 2D confinements, morphologies of block copolymers confined between a cylindrical pore as a function of surface field, pore radius and its thickness were also studied [10].

Compared to 1D and 2D confinements which have been well understood and documented, block copolymers in 3D confinements, on the other hand, have recently attracted considerable attention both experimentally [12–19] and computationally [20–25]. It is well known that in a confinement, the morphology of a block copolymer is affected by three main factors: (1) interactions between blocks, (2) interactions between blocks and surface boundary, and (3) the size of confinement i.e. the ratio between the size of the confinement space and the period of the domain in the bulk. Under these different conditions, block copolymers in 3D confinements produce a rich array of morphologies in the form of onion-like concentric lamellae, tennis balls, mushrooms, wheels, screw-like, stalked toroids and helices. Practically, those nanoparticles can be made in solutions or in spherical cavities. The forming of morphologies of block copolymers in solution depends on not only the polymer composition or the total degree of polymerization but also the polymer concentration, the nature of the common solvent, water content in solution, and the presence of additives such as homopolymers. For pure block copolymers in solution, spherical micelles are formed when the copolymer concentration is low, however, when the copolymer concentration increases the micelles change progressively from spheres to long rods with uniform diameter, to interconnected rods, and then vesicles [26, 27]. By adding a homopolymer onto a diblock copolymer solution [28] a phase transition from vesicle to spherical was obtained. Details of the relationship between diblock copolymer morphologies and solution concentration was also presented by Higuchi et al. using self-organised precipitation method [16, 17]. In those work, different morphologies such as lamellar, onion-like and hexagonally packed cylinders of a diblock copolymer particle as a function of solution concentration were obtained.

From the computational modelling perspective, a limited number of work has been carried out for block copolymers in 3D spherical confinements [20–25]. Yang et al. investigated the effect of an addition of a homopolymer on a copolymer/homopolymer blend confined in a spherical cavity. Depending on the volume fraction of the homopolymer, spherical pore surface properties and the composition of the diblock copolymer, many interesting morphologies in the forms of stacked toroids, helices and Janus-like of a nanoparticle were observed [22]. Those morphologies, as a function of surface fields and confinement space diameter, for a diblock copolymer confined in a spherical pore were also obtained using different computational methods [20, 21, 23]. Using self-consistent field simulations, Fraaije et al. have shown different bicontinuous structures in dispersed droplets of polymer surfactant [25]. However, in that work, details of effects of a presence of solvent in the droplet was not presented.

In our present work, the focus is on the effect of the homopolymer molecular weight on the morphology of an *A*–*B* diblock copolymer spherically confined by the homopolymer *C*. In contrast to earlier mentioned research for both hard and soft surface confinements which had the diblock copolymer completely confined within the boundary. In our system, however, the confinement depends on its environment, that the homoplymer is allowed to penetrate into the regions occupied by the diblock copolymers and changing the nature of its behaviour. Different morphologies of the structure are obtained by changing the diblock copolymer composition, the total volume fraction of the diblock, the homopolymer molecular weight and the interaction between the copolymer and the homopolymer. The effects of those parameters on the phase separation of a diblock copolymer/homopolymer system were previous carried out both experimentally [29] and computationally [30–32], but started from a homogeneous phase. In our calculation there are two main steps. The first step is to create a spherical domain that contains a disordered diblock copolymer spherically confined by a homopolymer. The second step focuses on the disorder-order phase transition under effects of a homopolymer presence in the sphere which strongly depends on its molecular weight and the interaction between the homopolymer and the diblock copolymer.

## Calculation methods

In the self-consistent field theory (SCFT) [33–37], a polymer is composed by subchains and each subchain is modeled by a linear flexible string made by a sequence of *N* segments. The segment is the primitive unit that constitutes the system. The spatial concentration distribution of the segment at position **r** is calculated through the statistical weight which follows the Edward equation:

1 $$\begin{aligned} \frac{\partial }{\partial s}Q_{i}(0,\mathbf{{r}}_{0};s, \mathbf{{r}}) = \left( \frac{b^2}{6}\nabla ^2 - \frac{1}{k_{B}T}V_{i}(\mathbf{{r}})\right) Q_{i}(0,\mathbf{{r}}_{0};s, \mathbf{{r}}). \end{aligned}$$

The path integral $Q_{i}(0,\mathbf{{r}}_{0};s, \mathbf{{r}})$ in Eq. 1 is defined as the sum of the statistical weights for all the conformations of the subchain *i* which has both ends, at the 0*th* and *N* *th* segments, fixed at positions $\mathbf{{r_{0}}}$ and $\mathbf{{r_{N}}}$. The segment density of the *i* *th* subchain at position $\mathbf{{r}}$ is calculated as [35]:

2 $$\begin{aligned} \phi _{i}(\mathbf{{r}}) = C \int q_{i}(s,\mathbf{{r}})\tilde{q}_{i}(N_{i} - s, \mathbf{{r}}) ds, \end{aligned}$$

with the integral is taken for the whole subchain *i*. *C* is the normalization constant. The forward and backward path integrals $q_{i}$ and $\tilde{q_{i}}$ are:

3 $$\begin{aligned} q_{i}(s,\mathbf{{r}}) = \int Q_{i}(0,\mathbf{{r}}_{0}; s,\mathbf{{r}}) d\mathbf{{r}}_{0}, \end{aligned}$$

and

4 $$\begin{aligned} \tilde{q}_{i}(N_{i} - s,\mathbf{{r}}) = \int Q_{i}(N_{i},\mathbf{{r}}_{N_{i}}; s,\mathbf{{r}}) d\mathbf{{r}}_{N_{i}}. \end{aligned}$$

For simplicity, both ends of a subchain are free ends, therefore the initial statistical weights at these free ends are unity. In Eq. 1, *b* is called the effective bond length—the length of the segment, $k_{B}$ is the Boltzman constant, *T* is the temperature and $V_{i}(\mathbf{{r}})$ is the mean field—the external potential acting on the subchain *i*. The self-consistent potential $V_{i}(\mathbf{{r}})$ is given by:

5 $$\begin{aligned} V_{i}(\mathbf{{r}}) = \sum _{K}\epsilon _{KK'}\phi _{K}(\mathbf{{r}}) - \mu _{i}(\mathbf{{r}}), \end{aligned}$$

with the summation is counted for all type of the segments, *K*, in the system. The segment–segment interaction parameter $\epsilon _{KK'}$ is determined from the Flory–Huggins interaction parameter, $\chi$, as:

6 $$\begin{aligned} \chi = \frac{z}{k_{B}T}\left\{ \epsilon _{KK'} - \frac{1}{2}(\epsilon _{KK} + \epsilon _{K'K'})\right\} , \end{aligned}$$

here *z* is the number of nearest neighbour sites.

The chemical potential in Eq. 5, $\mu _{i}(\mathbf{{r}})$, is calculated as a first derivative of the free energy respect to $\phi _{i}(\mathbf{{r}})$, where the free energy is given by [33, 35]:

7 $$\begin{aligned} \begin{aligned} F[\{\phi _{K}\},\{V_{K}\}] =&-k_{B}T\sum _{p}M_{p}ln Z_{p} + \frac{1}{2}\sum _{K}\sum _{K'}\int \epsilon _{KK'}\phi _{K}(\mathbf{{r}})\phi _{K'}(\mathbf{{r}}) d\mathbf{{r}} \\&- \sum _{K}\int V_{K}(\mathbf{{r}})\phi _{K}(\mathbf{{r}})d\mathbf{{r}}, \end{aligned} \end{aligned}$$

with $M_{p}$ being the total number of chains of p-type polymer and $Z_{p}$ being the partition function.

The statistical weights, segment density, chemical potential or free energy and mean field potential described in Eqs. 1, 2, 5 and  7, are related to each other and need to be solved iteratively [37].

## Model

The system we use throughout our calculation comprises a diblock copolymer *AB* mixed with a homopolymer *C*. Each diblock copolymer chain consisting of $N_{A}$ segments of *A*-type and $N_{B}$ segments of *B*-type. Here we choose the diblock chain length $N_{A} + N_{B} = 4 + 8 = 12$. This chain length is not too long to cause unnecessary cost of CPU time consumption and not too short to prevent microphase segregation taking place [31]. The homopolymer chain length, $N_{C}$, is chosen as $N_{C} = 1, 2$ and 5 (in segment unit). A real space simulation box of $28\times 28\times 28$ (in units of segment size that is taken to be unity) is divided into a grid mesh of $56\times 56\times 56$. To avoid the size-effect, we carried out different calculations of different box sizes, and from the results obtained for the free energy as a function of the box size we see that the box size from 28 $\times$ 28 $\times$ 28 would give us stable and accurate results. The spatial mesh width are chosen as $\Delta x = \Delta y = \Delta z = 0.5$, and the mesh size along the chain is chosen as $\Delta s = 0.2$ (for smaller values of $\Delta s$ we obtain the same result, however, the total CPU time is significantly increased when $\Delta s$ is decreased). The periodic boundary conditions are applied. A canonical ensemble, which keeps the total volume fraction of each polymer type in the system constant, is used. The total volume fraction of the diblock copolymer *AB* in the system is chosen at 10$\%$ and 20$\%$. This means that in the system there is 10$\%$ (or 20$\%$) of the diblock copolymer and 90$\%$ (or $80\%$) of the homopolymer. For the purpose of this work, the SUSHI code [37], which we use to perform SCFT simulations, is implemented throughout the investigation.

## Results and discussion

### Creating a spherical domain

**Fig. 1:**
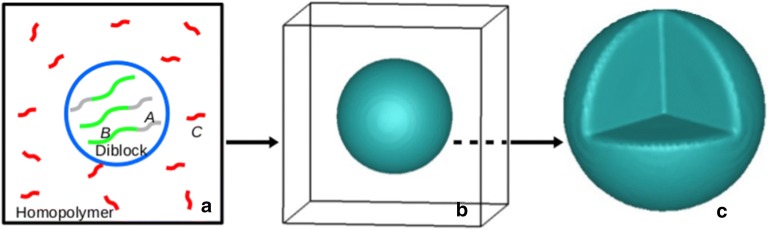
**a** A simulation box contains a diblock copolymer *A*–*B* and a homopolymer *C*. **b** A spherical domain that comprises of a diblock copolymer $A_{4}B_{8}$ after a domain confinement calculation was performed. The homopolymer that occupies the rest of the simulation box is not shown here. **c** A cut-through of the sphere

We start the calculation from a homogeneous system that contains $90\%$ of homopolymer and $10\%$ of diblock copolymer. A confinement method to confine the diblock copolymer in the middle of the box is implemented. In the first iteration step of the SCFT calculation we limit only the diblock to be in the middle of the simulation box. A small domain of the diblock in the middle of the box acting as a “seed” for the growth of a spherical diblock copolymer domain [38]. To make it simple, throughout the calculation we chose the Flory–Huggins interaction parameters between the homopolymer and diblock copolymer as $\chi _{AC} = \chi _{BC} = \chi$. This means that the two blocks of the diblock copolymer *A*–*B* interact with the homopolymer *C* equally. Thus, there is no selective wetting of the diblock copolymer at the copolymer/homopolymer interface. To have a phase separation between the copolymer and the homopolymer, the interaction between them has to be strong enough. Here we choose $\chi _{AC} = \chi _{BC} = 2.0$. Initially, the interaction between segments of the diblock copolymer is set at $\chi _{AB} = 0$. The result after running a static SCFT calculation is shown in Fig. 1. In this figure we shown the isosurface for the diblock copolymer ($\phi _{A}$ + $\phi _{B}$), which has a spherical shape and located at the center of the box. For the rest of the box, i.e. the surrounding area, which is completely dominated by the homopolymer, is not shown here. By varying the interaction between the copolymer and homopolymer, $\chi$, and the homopolymer chain length, $N_{C}$, we see that the occupation of the diblock copolymer in the spherical domain is proportional to the increase of $\chi$ or $N_{C}$. With $N_{C}$ = 1, the occupation of the copolymer in the spherical domain is 60.6$\%$, 85.0$\%$ and 92.3$\%$, for $\chi$ = 1.0, 1.5 and 2.0, respectively. Outside the sphere the occupation of the copolymer is up to $3\%$, for $\chi$ = 1.0, and when the interaction is strong enough, $\chi \ge 1.4$, the presence of the copolymer is almost disappeared. It means that when the interaction between the copolymer and the homopolymer is strong enough, outside of the sphere is totally occupied by the homopolymer, and inside the sphere is dominated by the copolymer. However, with this short chain length of the homopolymer, inside the sphere, there is always presence of the homopolymer.

Increasing the homopolymer chain length to $N_{C} = 2$ the occupation of the copolymer in the sphere increases to 92$\%$, $98\%$ and $99\%$ when $\chi$ is 1.0, 1.5 and 2.0, respectively. Further increasing the homopolymer chain length to $N_{C} = 5$, we obtain a total macrophase separation between the copolymer and homopolymer; in the sphere it is totally ($100\%$) occupied by the copolymer and outside it is totally occupied by the homopolymer.

### Effect of the homopolymer chain length

In this section, starting from a structure as shown in Fig. 1b, we calculate the microphase separation of the diblock copolymer in the sphere for different homopolymer chain lengths. First, we use a short homopolymer chain length, $N_{C} = 1$. The interaction between the copolymer and the homopolymer is chosen at $\chi = 1.0$, we see that when the interaction between two segments *A* and *B* of the diblock, $\chi _{AB}$, increases from the initial zero value the occupation of the copolymer in the sphere is reduced. For example, the occupation of the copolymer is about $56\%$ when $\chi _{AB} = 0.2$, but when $\chi _{AB}$ increases to 0.4 the occupation reduces to $50\%$. This is due to the fact, that for a short homopolymer chain length and the interaction between the homopolymer and the copolymer is weak, inside the sphere always has a presence of the homopolymer and when the repulsive interaction between monomers *A* and *B* increases the homopolymer migrates into the interface of *A* and *B* domains. Furthermore, at this weak interaction regime between the copolymer and the homopolymer, $\chi = 1.0$, the homopolymer outside the sphere can easily move inside the sphere and dilutes the diblock, hence, reduces the interaction between segments and prevents the diblock from segregating. Keep increasing $\chi _{AB}$ to by 0.8 we observe that the whole system becomes homogeneous. This result is in well agreement with results obtained by Matsen [39] on the effect of the homopolymer molecular weight on the microphase transition in a weakly separated diblock copolymer and homopolymer blend. They found that at low weight homopolymers tend to be miscible with the microstructure, causing the lattice spacing to diverge and the system becomes homogeneous. This is because small homopolymers tend to distribute uniformly throughout the melt. Their nearly uniform distribution will produce a field with little spatial variation and thus with little tendency to induce segregation. They will instead dilute the copolymer concentration, effectively reducing the interaction between segments. The same conclusion was also made by Semenov [40] for diblock copolymer and homopolymer blends in a strong segregation regime. It is worth mentioning that when the homopolymer chain length is short the effect from the thermal fluctuation would be quite significant, however, in the case of short chain length the phase-separation only happens in a really low temperature regime hence the contribution from thermal fluctuation could be neglected [41].

**Fig. 2:**
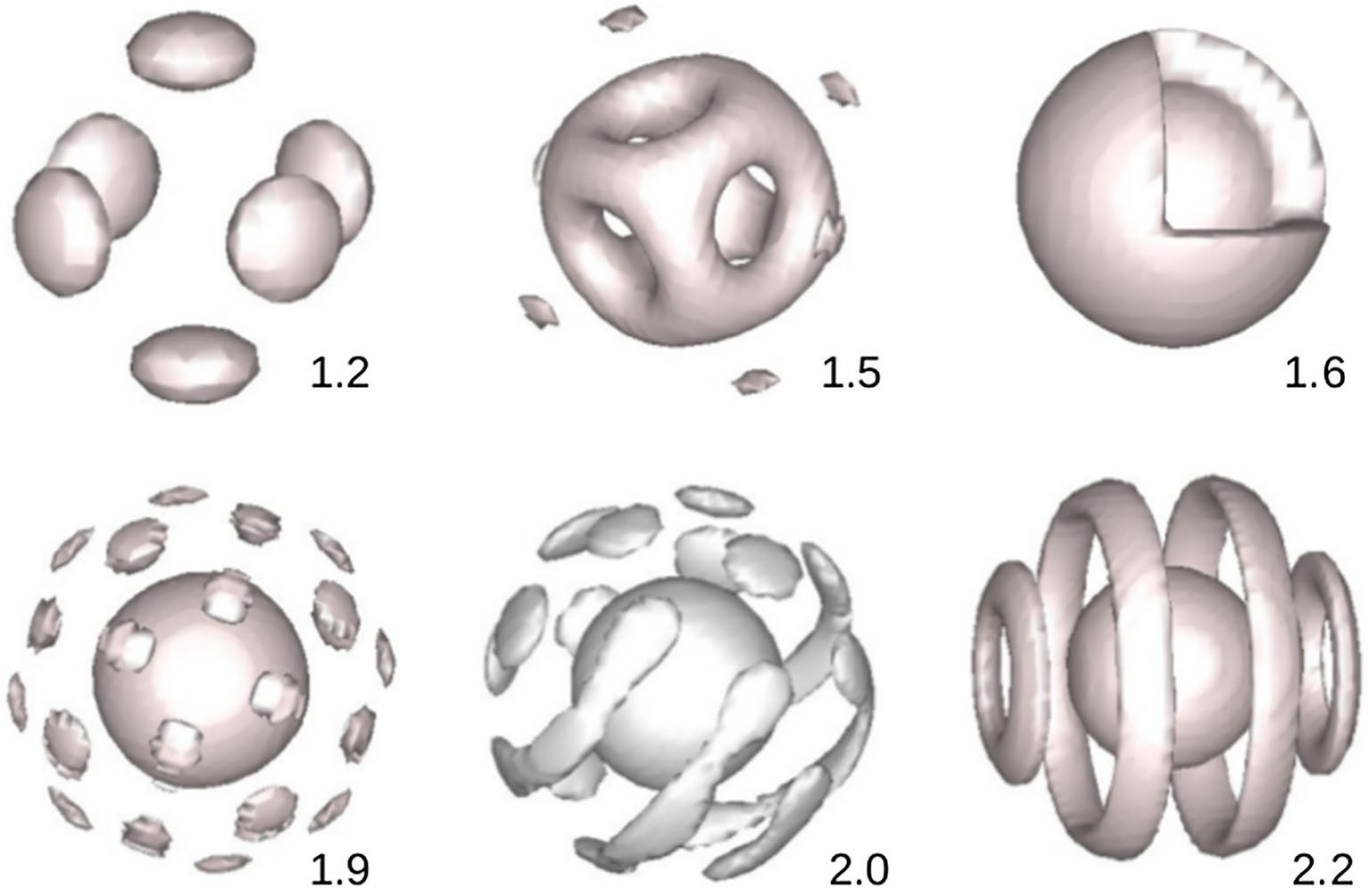
Morphology of the diblock copolymer as a function of the interaction between components *A* and *B* of the diblock, $\chi _{AB}$. The chain length of the homoplolymer, $l_{h} = 1$, the total volume fraction of the copolymer is $10\%$ and the interaction between the homopolymer and copolymer $\chi = 2.0$. Throughout the calculation we chose the interaction parameters between the diblock copolymer *A*-*B* and homopolymer *C* equally, $\chi _{AC} = \chi _{BC}$. Unless stated otherwise, thereafter the morphologies are shown for component *A* with the isosurface $\phi _{A} = 0.5$

In a weak interaction regime between the homopolymer and the copolymer, there is no microphase segregation between the diblock copolymer observed for any interaction value of $\chi _{AB}$. By increasing the interaction between the homopolymer and the copolymer, $\chi$, we see that the microphase starts to segregate, for example, at $\chi = 2.0$, in Fig. 2 we show different stable morphologies for the *A*-type polymer inside the sphere at different values of $\chi _{AB}$. At $\chi _{AB} = 2.0$, when the microphase segregation takes place, in the interface regions between *A*-rich and *B*-rich layers there is presence of the homopolymer, up to $18\%$ (of the volume fraction). Also, in the *A*-rich and *B*-rich domains themselves the occupations of the homopolymer are $5\%$ and $4\%$, respectively. It is also important to notice that by choosing the interaction between the homopolymer and copolymer strong enough, in the space outside the sphere it is totally occupied by the homopolymer and the copolymer is completely vanished.

**Fig. 3:**
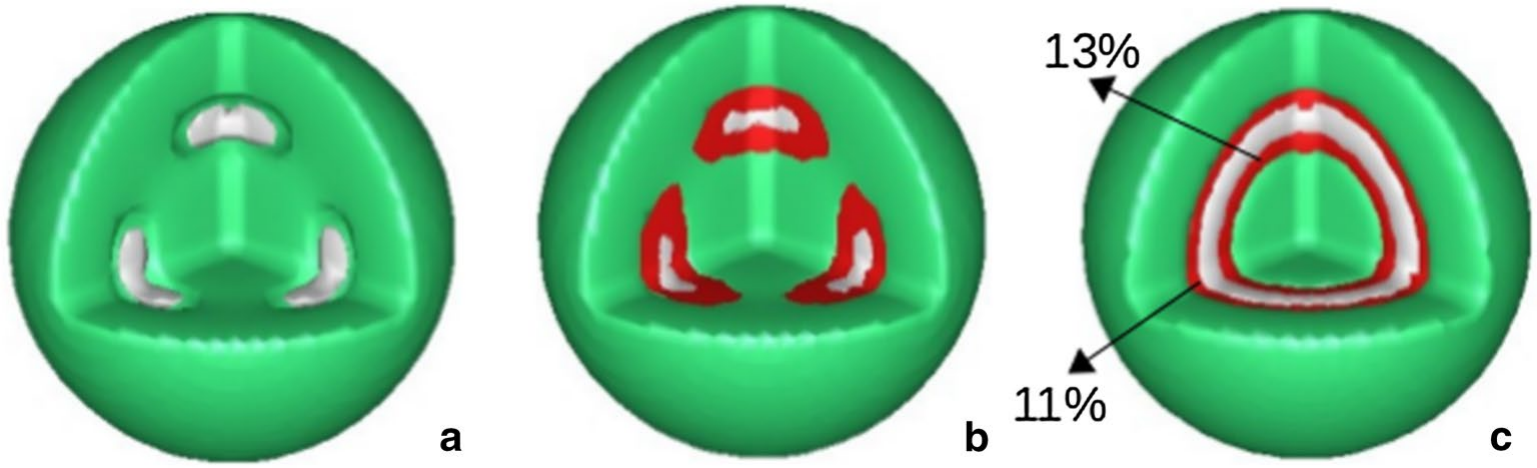
Isosurfaces of three components in the system, $\chi _{AB} = 1.2$ (**a**, **b**) and $\chi _{AB} = 1.6$ (**c**). *A*-component (gray), *B*-component (green) and homopolymer *C* in red

**Fig. 4:**
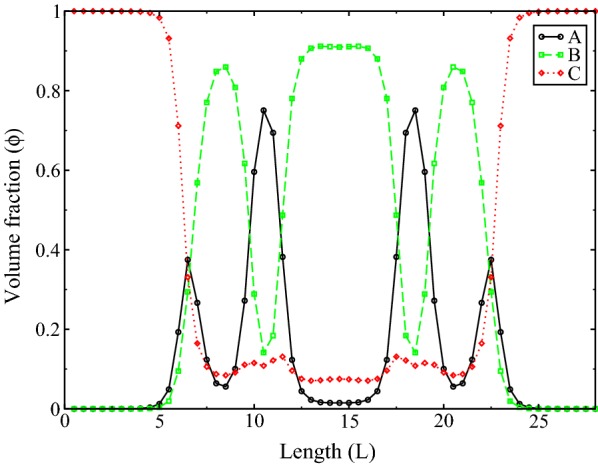
Changes of the volume fraction for all three monomer components A, B and C along a symmetrical line of the box, for the case of $\chi _{AB} = 1.6$ in Fig. 3

To make things clear, in Fig. 3 we show the morphologies for all the components in the system, grey colour represents *A*-type, green for *B*-type and red for homopolymer *C*. In Fig. 3a only two components *A* and *B* are plotted, with isosurfaces $\phi _{A} = \phi _{B} = 0.5$. From this picture we see that at the interfaces between *A*-rich and *B*-rich there is a gap. This gap is significantly filled with the homopolymer, up to 11$\%$ for the case of $\chi _{AB} = 1.2$. The plot for all three components is shown in Fig. 3b, with the isosurfaces $\phi _{A} = \phi _{B} = 0.5$ and $\phi _{C} = 0.1$. The structure for $\chi _{AB} = 1.6$ is shown in Fig. 3c, with the occupation of the homopolymer at the two interfaces is in the range of $11\%$ $\rightarrow$ $13\%$. To see how the polymers distribute in the system, in Fig. 4 we show volume fractions along a symmetrical line of the simulation box for all three components, for the case shown in Fig. 3c at $\chi _{AB} = 1.6$. From this figure we see that the homopolymer occupies throughout the space and its presence peaks at the interfaces between the diblocks.

The presence of the homopolymer in the sphere depends not only on its interaction with the diblock copolymer but also the homopolymer chain length. To see details how the homopolymer chain length affects the diblock phase separation we now increase the homopolymer chain length to $N_{C} = 5.0$. We observe that in the initial structure of the sphere when the interaction between monomers *A* and *B* ($\chi _{AB}$) was set at zero, the occupation of the copolymer in the sphere is almost $100\%$ even the interaction between the copolymer and homopolymer is weak, at $\chi = 1.0$. Some morphologies shown in Fig. 2 for $N_{C} = 1.0$ are also obtained for $N_{C} = 5.0$. However, in the latter the same morphology is obtained at a weaker value of $\chi _{AB}$ compared to the former. Furthermore, unlike the previous case where at $\chi = 1.0$ the system becomes homogeneous when the interaction between *A* and *B*, $\chi _{AB}$, is strong, in the latter case, however, we observe phase segregation for high values of $\chi _{AB}$.

### A bigger sphere

It has been shown in other works, that the morphology of a nanoparticle really depends on the size of the confinement [12, 20, 42]. To see how the size of the spherical domain affects its inner structures, we increase the diblock copolymer concentration in the simulation box from 10 to 20$\%$. It is worthnoting that by increasing the size of the diblock spherical domain, the size of the bulk homopolymer in a fixed simulation box is decreasing and this could lead to a size effect of “bulk” homopolymer which confines the diblock sphere. To make sure our chosen system size ($28 \times 28 \times 28$) is large enough in order to eliminate the size effect, we carried out calculations for different box sizes but kept the volume of the diblock copolymer spherical domain constant. Our results show that at the size box from $26 \times 26 \times 26$ all the results are identical.

**Fig. 5:**
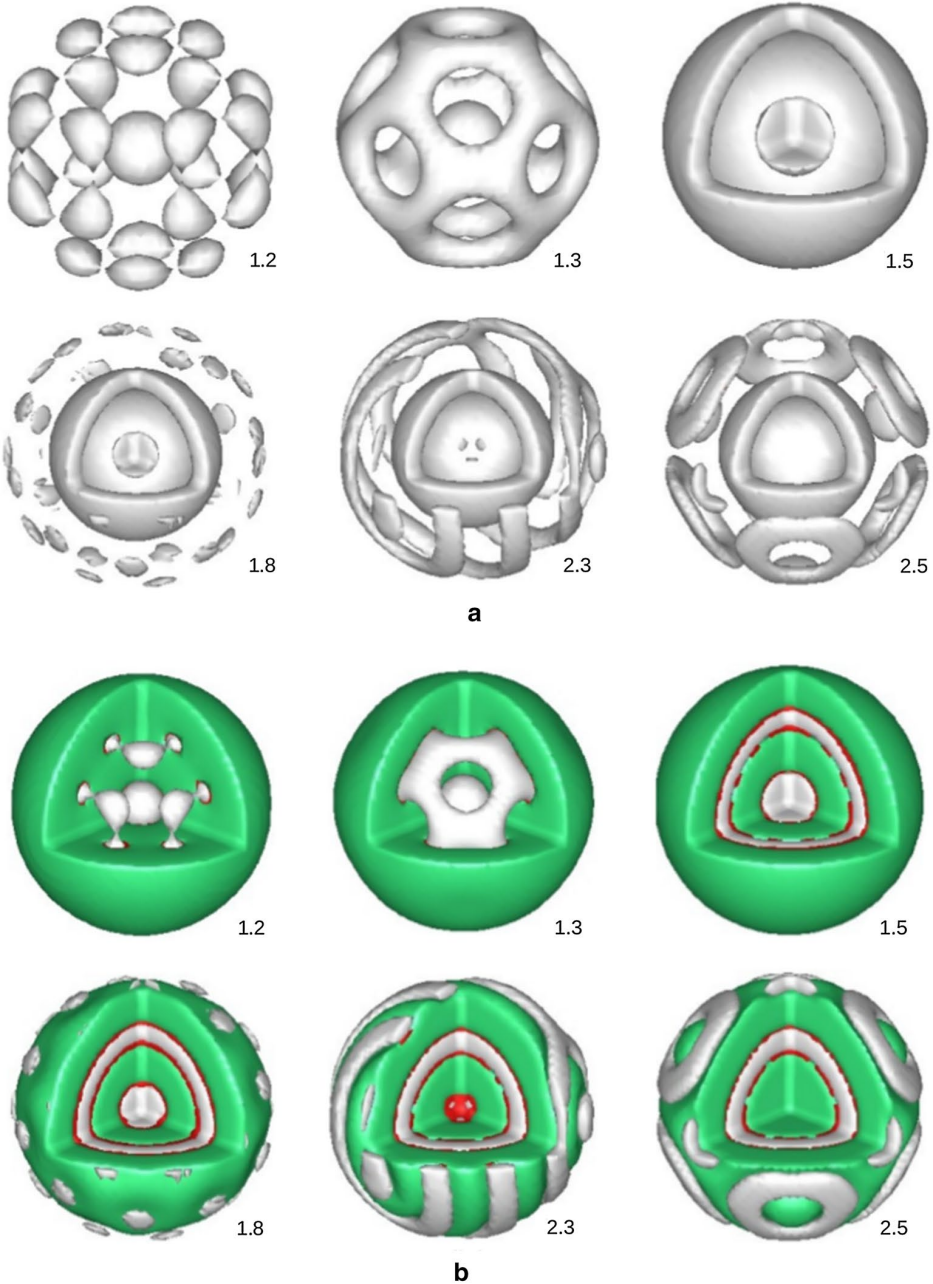
Morphology of the diblock copolymer as a function of $\chi _{AB}$ when the concentration of the diblock is increased to $20\%$. The homopolymer chain length $l_{h} = 1$, and the interaction between the copolymer and homopolymer is $\chi = 2.0$. **a** Plot for *A*-component and **b** plot for three components, *A*(gray), *B*(green) and *C*(red)

With a bigger spherical domain more morphologies are observed compared to the previous case of a smaller one. In Fig. 5 we show morphologies as a function of the interaction parameter $\chi _{AB}$. Figure 5a shows for the component *A*, and Fig. 5b shows for all three components *A*, *B* and *C*. Like the previous case, with $N_{C} = 1.0$ and $\chi = 2.0$, the microphase separation of the diblock takes place at $\chi _{AB}$ as small as 1.2. At $\chi _{AB} = 1.2$, in the center of the sphere forms a small spherical *A*-rich domain and in the outer layer forms 24 small islands. Increasing $\chi _{AB}$ to 1.3, these small islands grow and then connect to each other and form a cage which comprises of 14 holes. From $\chi _{AB} = 1.5$ the domains on the cage layer grow and fill all the holes to form an uniform shell. At $\chi _{AB} = 1.8$, apart from the uniform shell, there is a new outer layer. This new layer comprises of many small islands. Continue increasing the $\chi _{AB}$, these islands connect and form stripes, at $\chi _{AB} = 2.3$. From $\chi _{AB} = 2.5$ we have a coexisting of islands (6 islands) and discs (8 discs). In the center of the sphere it is now occupied by the *B*-rich domain, not *A*-rich domain like cases when $\chi _{AB}$ is smaller than 2.5. The coexisting of islands and discs remains until $\chi _{AB} = 2.8$ where the structure becomes a new uniform shell. Morphologies in Fig. 5 were also obtained for a diblock copolymer confined in a spherical surface under different surface fields and degree of confinement [15, 20, 23], and homopolymer volume fraction [22].

**Fig. 6:**
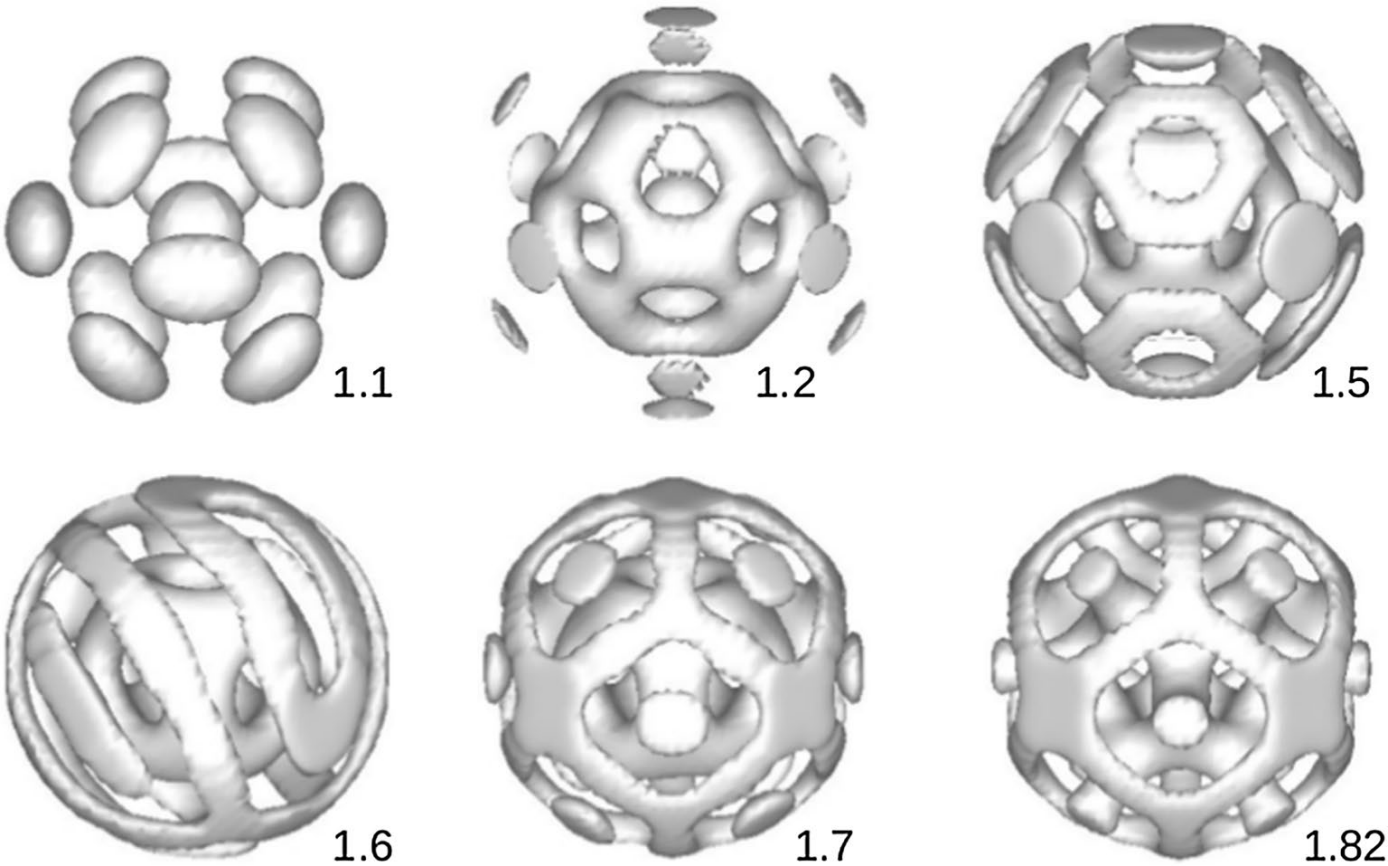
The same as Fig. 5 but for $l_{h} = 5$

Like the previous case of a smaller sphere, the presence of the homopolymer inside the sphere changes dramatically when the homopolymer chain length is increased. Indeed, by increasing the homopolymer chain length $N_{C}$ to 5.0 we obtain a complete macrophase separation, copolymer totally fills up the sphere and surrounded by the homopolymer. In this case our system can be compared with systems of diblock copolymers confined in a hard [23] or soft [15, 22, 43] spherical surface. Different morphologies when the homopolymer chain length $N_{C} = 5$, for different values of $\chi _{AB}$, are shown in Fig. 6.

## Conclusions

By using the static self-consistent field theory we have studied the microphase separation of a diblock copolymer spherically surrounded by a homopolymer. When the homopolymer chain length is short, in order to have a microphase separation in the diblock copolymer, the interaction between the diblock copolymer and the homopolymer needs to be strong. Because of a short chain length, the homopolymer can easily penetrate into spaces dominated by the copolymer and reduces the interaction between segments of the diblock and prevents them from segregating. When the diblock copolymer undergoes phase segregation, a significant amount of the homopolymer is observed to be occupying not only in the interface between *A*-rich and *B*-rich regions but also in the *A*-rich and *B*-rich regions themselves. The presence of the homopolymer in the sphere is significantly reduced when the homopolymer chain length is increased. When the homopolymer chain length is long enough a total macrophase separation between the homopolymer and the diblock copolymer is obtained. Inside the sphere, depending on the interactions between monomers *A* and *B*, and between the homopolymer and the copolymer, different morphologies are obtained such as islands, cage, stripes, uniform shell and branch are achieved. It was our aim, that this work—the methodology and computational modelling—could provide a new method of controlling the assembly of matter with sufficient certainty and precision to allow the preparation of materials and molecular assemblies, with far more sophisticated and tuneable properties and functions than are accessible in materials synthesised using current traditional methods [2]. Understanding the mechanism of creating block polymer-based nanoparticles and how to switch phase between different morphologies is a very important step prior to developing a new hybrid method accommodating for both inorganic and organic nanoparticles. Furthermore, understanding the mechanism of phase transition in soft matter systems could help to develop new effective techniques in food processing which can create food with different sensory properties, product stability and visual impressions. As food can be regarded as multi-components mixtures whose components have a large different in length scale, physical and chemical properties [44]. By tailoring their length scale and correlation interaction between components different phases such as liquid, crystalline, foams and emulsions can be obtained [45, 46]. Looking to the future, in our on going work, we are developing a method of encapsulating and releasing metallic nanoparticles using block copolymer-based nanocontainers which can by applied as drug delivery vehicles, nanoreactors, fuel cell and photovoltaic.
